# Sea Voyages

**Published:** 1867-12

**Authors:** 


SEA VOYAGES, and the best means of enjoying them; and first of all have a plenty of woolen clothing and wear it even in midsummer except during the middle of still days ; but every day, and all day a good flannel shirt should be worn next the skin even in the tropics, to counteract the baleful effect of damps, fogs, and changes of temperature. The British government compels its sailors to wear woolen flannel shirts all the year round in all latitudes as a result of its observed necessity in keeping off disabling diseases.

PROTECTING the FEET

from the dampness of the decks is an indispensable item of health and comfort on ship-board as the boards are seldom dry for two hours at a time in any voyage. Thick soled shoes and woolen hose should be worn at all times while at sea. Much has been said and written in praise of the

